# Antiproliferative, antibacterial, and antioxidant activities of *Bauhinia strychnifolia* Craib aqueous extracts in gut and liver perspective

**DOI:** 10.1186/s12906-021-03448-2

**Published:** 2021-11-04

**Authors:** Suranat Phonghanpot, Faongchat Jarintanan

**Affiliations:** 1 Biochemistry Unit, Department of Biomedical Science, Faculty of Sciences, Rangsit University, 52/347 Muang Ake, Phaholyothin road, Lak Hok, Muang, Pathum Thani, 12000 Thailand; 2 Faculty of Medical Technology, Rangsit University, 52/347 Muang Ake, Phaholyothin road, Lak Hok, Muang, Pathum Thani, 12000 Thailand

**Keywords:** Thai traditional medicine, Herbal medicine, Antiproliferation, Antimicrobial, Hangover remedy

## Abstract

**Background:**

*Bauhinia strychnifolia* Craib is an herb in Thai traditional medicine. Its decoction is traditionally used as an anticancer, antidiarrheal, and hangover remedy for centuries. Several studies described bioactivities of its organic solvent extracts, however, only few demonstrated the usefulness of the decoction. Here, we aimed to determine the bioactivities of *Bauhinia strychnifolia* Craib root and stem aqueous extracts in gut and liver perspective.

**Methods:**

To achieve the goal, we performed MTT test, microscopic analyses, disc diffusion assay, broth microdilution assay, free radicals scavenging assays, and LC-MS analysis.

**Results:**

We found that the extracts inhibited the growth of human hepatocellular carcinoma (HepG2) and colon adenocarcinoma (HT-29) cell lines. Moreover, they also inhibited the growth of gram-positive bacteria *Staphylococcus aureus* and *Bacillus cereus* but not inhibited the growth of gram-negative bacteria *Escherichia coli* and *Pseudomonas aeruginosa*. Furthermore, the extracts exhibited moderate antioxidant activity and increased GSH production in HepG2 cell line when compared with untreated. Our LC-MS analysis confirmed the existence of anticancer and antioxidant; 3,5,7,3′,5′-pentahydroxyflavanonol-3-O-α-L-rhamnopyranoside and β-sitosterol, in the extracts.

**Conclusion:**

The results from our study supported that the administration of *Bauhinia strychnifolia* Craib root and stem decoction would really aid colon or liver cancer patients and detoxify the alcoholic drunkard as it is claimed in Thai traditional medicine.

**Supplementary Information:**

The online version contains supplementary material available at 10.1186/s12906-021-03448-2.

## Background

*Bauhinia strychnifolia* Craib is a climbing herb and a member of Caesalpiniaceae that have been used in Thai traditional medicine [1]. The young stem is brown grey in colour and changes toward red brown within the maturation. Length of the mature stem can be found at average of 5 m. Rough root of *Bauhinia strychnifolia* Craib is usually seen in black but the inside is red brown. *Bauhinia strychnifolia* Craib sprouts cluster of flowers at the end of its stem, which can be seen during May to August. We can grow *Bauhinia strychnifolia* Craib by several methods, such as seeding, grafting, and clustering. *Bauhinia strychnifolia* Craib grows very fast on loam and needs moderate amount of water together with strong sunlight [2, 3].

A decoction prepared from *Bauhinia strychnifolia* Craib contains several beneficial medical properties and mainly used as anticancer, antidiarrheal, antihistamine, anti-inflammation, antimalarial, fever remedy, hangover remedy, and insecticide antidote [3–7]. Considering anticancer activity, results from a study showed that *Bauhinia strychnifolia* Craib dichloromethane and ethanol extracts possess strong anticancer activity against A549 (lung), KB3–1 (cervical), MDA-MB-231 (breast), and SW480 (colon) cancer cell lines via MTT assay [4]. More recent, there was a study isolating chemical constituents from *Bauhinia strychnifolia* Craib root and stem ethanol extracts and analyzing for their anticancer activity. They successfully isolated 5 compounds, which exhibit cytotoxic activity against HeLa (cervical), HT-29 (colon), KB (oral), and MCF-7 (breast) cancer cell lines [3]. Latest in 2016, leaf ethanolic extracts of *Bauhinia strychnifolia* Craib were also analyzed for their anticancer activity against KKU-M156 (bile duct), LS174T (colon), and SW480 (colon) cell lines. The results showed that the vacuum liquid chromatography fraction of the ethanolic extracts could kill the mentioned human cancer cell lines [6].

Phytochemical study of the decoctions, which are prepared from different parts of the *Bauhinia strychnifolia* Craib, revealed the presence of several bioactive compounds including alkaloids, flavonoids, phenolic compounds, saponins, steroids, terpenoids, and triterpenes [8]. Administration of *Bauhinia strychnifolia* Craib decoction would aid the consumers gut to blood ecosystems consisting of inhibition of cell division, reduction of pathogenic microbes, and detoxification of free radicals. According to the traditional administration, Thai people in the Northeast of Thailand prepare *Bauhinia strychnifolia* Craib as a decoction rather than ethanol beverages [2, 3, 5–7]. However, the majority of the previous studies described the activities of organic solvent extracts of *Bauhinia strychnifolia* Craib against cancer cell lines. There were only few reports existing to explain the usefulness of *Bauhinia strychnifolia* Craib aqueous extract or its decoction. In this work, we aimed to analyze the impact of *Bauhinia strychnifolia* Craib root and stem aqueous extracts against colon and liver cell lines as a model of the traditional administration. To achieve the goal, we determined antiproliferative effect of the aqueous extracts against HepG2 and HT-29 cell lines, antibacterial activity against 4 major guts pathogenic bacterial strains, and antioxidant activity. The results of our study provided deeper understanding toward the benefits of *Bauhinia strychnifolia* Craib decoction.

## Methods

### Preparation of *Bauhinia strychnifolia* Craib aqueous extracts and samples

In this experiment, we obtained root and stem *Bauhinia strychnifolia* Craib aqueous extracts from Assoc. Prof. Dr. Surapot Wongyai (Lecturer, Faculty of Oriental Medicine, Rangsit University). The plant materials were harvested, compared, and identified with the voucher specimen number BK No. 084965 (kept at BK Herbarium, Botanical Section, National Department of Agriculture, Thailand) by the harvester, Nirun Vipunngeun (Lecturer and Botanist, Department of Pharmacognosy, Faculty of Pharmacy, Rangsit University) from the Faculty of Pharmacy, Rangsit University’s cultivation field before using in extract preparation. The extracts were prepared by boiling approximately 1 kg of the grinded root and stem in 5 L of water. Consequently, the filtered aqueous solutions were evaporated to obtain powder extracts using evaporator. For antibacterial and antioxidant analysis, the extracts were quantitated and dissolved in distilled water at different concentrations. For MTT and microscopic assays, the extracts were dissolved in DMEM (Gibco, USA) with 0.1% DMSO to obtain the desired final concentrations. The extracts were dissolved with methanol to the final concentration of 10 mg/mL for LC-MS analysis. Consequently, the samples were filtered through 0.25-μm filters before subject 10 μL of each into the machine.

### Determination of antiproliferative activity by MTT assay

We used MTT assay to determine antiproliferative activity of the extracts against human hepatocellular carcinoma (HepG2 [HEPG2], ATCC HB-8065) and colon adenocarcinoma (HT-29, ATCC HTB-38) cell lines. We obtained the cell lines in collaboration with Asst. Prof. Dr. PotJanee Stimanote (Graduate Program in Biomedical Sciences, Faculty of Allied Health Science, Thammasat University) who purchased the cell lines from ATCC (ATCC, USA). We maintained the cell lines in DMEM, supplemented with 10% FBS (Gibco, USA), 100 U/mL of penicillin-streptomycin (Gibco, USA). Following the standard protocol, in a 96-wells plate, approximately 5 × 10^4^ cells were seeded and incubated 24 h at 37 *°*C in an atmosphere of humidified air with 5% CO_2_. The medium in each well was then removed, replaced with the new medium containing the final concentrations of crude extract, and incubated for 24 more hours. For vehicle control, 0.1% DMSO was used instead of the extracts. We then added 100 μL of 2.5 mg/mL MTT solution into each well and incubated for 1 h at 37 °C. The purple formazan crystal were dissolved by addition of 100 μL DMSO. Consequently, we read the absorbance at 570 nm using microplate reader. The percentage of surviving cells was calculated as % cell viability [9, 10]. Dose-response curves were constructed to achieve the IC_50_ values. All experimental data were derived from 3 independent experiments.

### Phase contrast microscopic assay

To investigate the effect of root and stem aqueous extracts toward HepG2 and HT-29 cell lines morphological changes, phase contrast microscopic analysis was performed. Briefly, 5 × 10^4^ cells were seeded into 96-wells plate and incubated overnight. After that, the cell lines were treated with the root and stem extracts at their IC_50_ concentrations and incubated for 24 h. For negative control, 0.1% DMSO was used instead of the extracts. The morphology of the cell lines were then examined using phase contrast inverted microscope at 10X magnification.

### Hoechst 33342 staining

We examined the nuclei morphological changes of Hoechst 33342 stained HepG2 cells using fluorescence-inverted microscope. At the beginning, the HepG2 cells were plated at 1 × 10^5^ cells/mL in 4-well chamber slide (Lab-Tek, USA) and incubated for overnight. The cells were treated with the root and stem extracts at their IC_50_ concentration and incubated for 6 and 12 h. The negative (untreated) and positive (drug treated) controls were treated with 0.1% DMSO and 5 μM doxorubicin, respectively. The cells were washed with PBS and incubated with 5 μM Hoechst 33342 for 30 min in the dark at room temperature. The cells were then washed 3 times with PBS before examining their nuclei morphology under fluorescence-inverted microscope at 10X magnification.

### Disc diffusion assay

In this work, we used *Bacillus cereus* ATCC14579, *Escherichia coli* ATCC25922, *Pseudomonas aeruginosa* ATCC27853, and *Staphylococcus aureus* ATCC25923 (ATCC, USA) as gut pathogenic representatives for screening of antibacterial activity of the extracts using disc-diffusion and broth microdilution assays. For disc diffusion assay, a single colony of each bacteria was selected, sub-cultured into 5 mL NB (HiMedia, India), and incubated for 24 h at 37 *°*C. Each culture was then added into NSS until the turbidity reach McFarland’s standard No. 0.5. The inoculated NSS (approximately 1.5 × 10^8^ cells/mL) was swabbed on freshly prepared NA. Sterile 6 mm diameter filter paper discs (Whatman, UK) were impregnated with 2 and 5 mg extracts before placing on the bacterial swabbed NA. After the incubation at 37 *°*C for 24 h, the antibacterial activity was evaluated by measuring the IZD. We used ampicillin (10 μg/disc), ceftriaxone (30 μg/disc), chloramphenicol (30 μg/disc), and rifampicin (5 μg/disc) (Oxoid, UK) as positive controls for inhibition of *Escherichia coli*, *Pseudomonas aeruginosa*, *Bacillus cereus*, and *Staphylococcus aureus*, respectively.

### Broth microdilution assay

According to the positive results from disc diffusion assay, the MIC values of the extracts against *Bacillus cereus* and *Staphylococcus aureus* were determined by a service of Faculty of Pharmacy, Rangsit University. Following standard protocol with few adaptation, 100 μL of two-fold serial dilutions of the root and stem aqueous extracts were prepared using MHB into 12 concentration ranging from 1028 to 0.5 μg/mL in 96-wells plate. After that, 10 μL of bacterial suspension (approximately 1.5 × 10^8^ cells/mL comparing with McFarland’s standard No. 0.5) was added into each well. The plain medium wells without bacteria or bacterial wells without extract were also included in the plates serving as blanks and negative controls, respectively. For positive control, chloramphenicol and rifampicin were used instead of the extracts against *Bacillus cereus* and *Staphylococcus aureus*, respectively. Consequently, we cultured the plates at 37 °C for 18 h before determining the MIC values by the unaided eye.

### ABTS radical scavenging assay

Total antioxidant capacity measurement of the crude extracts was carried out using the Cayman’s antioxidant assay kit (Cayman, USA). Following the manufacturer’s protocol, 10 μL of each Trolox standard, 10 μL of metmyoglobin, and 150 μL of chromogen was added into the provided 96-wells plate for duplication. For the sample wells, 10 μL of 100 mg/mL root or stem extracts, 10 μL of metmyoglobin, and 150 μL of chromogen were added into the plate for triplication. The reaction was initiated by addition of 40 μL of H_2_O_2_ working solution into each well that was being used. After covering the plate with the plate cover, the plate was shaken and incubated for 5 min in room temperature. We, then, read the absorbance of the mixtures at 750 nm. Antioxidant capacity of the extracts was calculated into μmol/g Trolox equivalent using standard calibration curve.

### Determination of total GSH

The colorimetric GSH + GSSG/GSH assay kit (Abcam, USA) was used for evaluating GSH amount inside the extracts treated HepG2 cell line. According to the manufacturer’s instructions, approximately 8 × 10^5^ HepG2 cells were seeded into each well of 6-wells plate. The cells were pretreated with 0.05 or 0.10 mM H_2_O_2_ for 2 h following by the treatment of stem (0, 0.15, and 0.3 mg/mL) or root (0, 0.3, and 0.6 mg/mL) extracts for 24 h. After harvesting the cells, they were washed with ice-cold PBS, centrifuged to remove supernatant, and lysed with GSH buffer. After 10 min incubation, 5% SSA was added, centrifuged, and then collected the supernatant further analysis. The 160 μL of reaction mix solution was added to each well of 96-wells plate and incubated for 10 min. After that, we added 20 μL of either the GSH standard solutions or the samples into each well and incubated in room temperature for 10 min. Finally, the 20 μL substrate solution was added into each well and incubated at room temperature for 10 min. Consequently, we determined the absorbance at 405 nm using microplate reader. Concentration of GSH in the sample was calculated by linear regression with the standard GSH calibration curve.

### Liquid chromatography tandem mass spectrometry (LC-MS) analysis

We analysed the chemical composition inside the root and stem aqueous extracts by the service of RSU Scientific and Technological Research Equipment Center (Rangsit University, Thailand). To separate the chemical constituents of the extracts, we used Vertisep reverse phase column C18 (Vertichrom, Thailand) equipped with UltiMate 3000 UHPLC systems (Thermo Scientific, USA). Using mobile phase as water (A) and acetonitrile (B) with flow rate of 0.3 mL/minute, the following step gradient condition was applied for liquid chromatography operation: 0–20 min 5% B; 20–30 min from 5 to 50% B; 30–50 min 50% B; 50–60 min from 50 to 80% B; 60–80 min 80% B; 80–90 min from 80 to 100% B; 90–120 min 100% B; 120–140 min from 100 to 5% B; and the column was equilibrated for 140–155 min with 5% B before the next run. For electrospray ionization - ion trap mass spectrometry, we used amaZon SL systems (Bruker, USA) with the following parameters: capillary voltage + 4500 V, source temperature 220 °C, Helium desolvation gas flow 7 L/minute, and nebulizer pressure 2.0 bar. For full scan MS analysis, the spectra were recorded in the negative mode in the range of m/z 200–500.

### Statistical analyses

In general, the independent triplicate assay values were expressed in the form of mean ± SEM. We used one-way ANOVA following by Tukey HSD and Friedman tests to confirm the significant differences between DMSO control group with root and stem aqueous extract groups in all assays. When the *p*-value < 0.05, they were considered as statistically significant. All of the statistical analyses were performed using Microsoft Excel, Statistics Kingdom, and Statology online tools (https://www.statskingdom.com, https://www.statology.org).

## Results

### Antiproliferative activity of *Bauhinia strychnifolia* Craib aqueous extracts against the growth of HepG2 and HT-29 cell lines

**Fig. 1:**
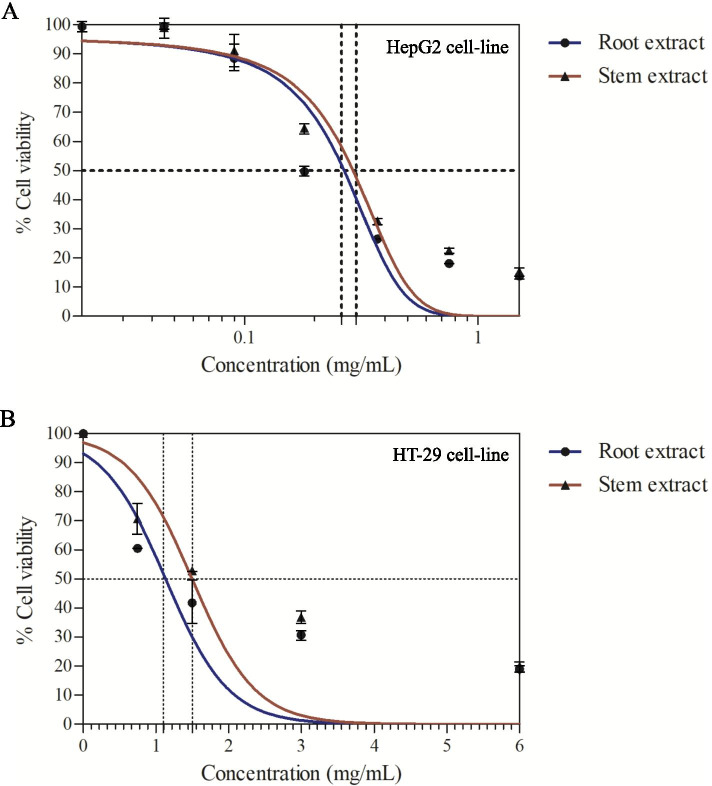
Graphical plot of aqueous extracts concentrations and calculated % cell viabilities relative with control untreated cell lines. **A** MTT test of root and stem aqueous extracts against HepG2 cell line. **B** MTT test of root and stem aqueous extracts against HT-29 cell line

We determined the antiproliferative activity of the *Bauhinia strychnifolia* Craib root and stem aqueous extracts against HepG2 and HT-29 cell lines, which represent hepatocellular carcinoma and colon adenocarcinoma, respectively. According to the different sensitivity of the cell lines to the extracts, the final concentrations of each extract against each cell line in this work were 0 to 1.50 mg/mL and 0 to 6.00 mg/mL for HepG2 and HT-29 cell lines, respectively. Using MTT assay, the % cell viabilities of each extract against each cell were calculated and plotted in graphical representation. Obviously, the root and stem aqueous extracts of *Bauhinia strychnifolia* Craib reduced proliferation of HepG2 and HT-29 (Fig. 1). IC_50_ values of root and stem extracts against the growth of HepG2 cell line were 0.22 ± 0.01 (*p*-value < 0.001) and 0.30 ± 0.01 (*p*-value < 0.001) mg/mL, respectively. In addition, IC_50_ values of root and stem extracts against the growth of HT-29 cell line were 1.13 ± 0.38 (*p*-value < 0.001) and 1.50 ± 0.32 (*p*-value < 0.001) mg/mL, respectively (Table 1).

**Table 1:** IC_50_ of root and stem aqueous extracts against HepG2 and HT-29 cell lines

Cell line	IC_50_ (mg/mL) (mean ± SEM)	
	Root extract	Stem extract
HepG2	0.22 ± 0.01	0.30 ± 0.01
HT-29	1.13 ± 0.38	1.50 ± 0.32

### Observation of apoptosis liked behavior by microscopic analysis

**Fig. 2:**
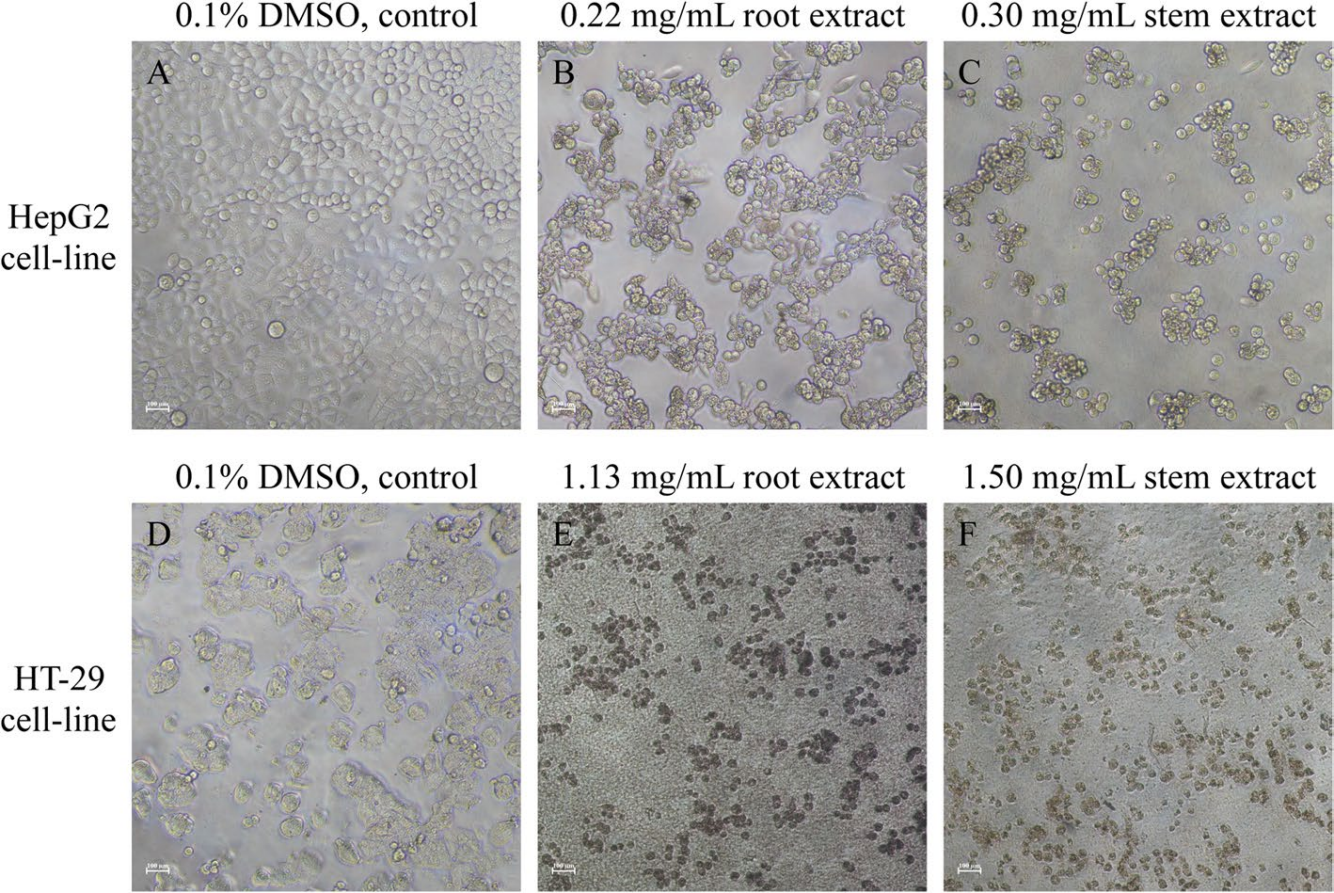
Phase contrast microscopic analysis of extracts treated HepG2 and HT-29 cell lines. **A** HepG2 cell line treated with 0.1% DMSO. **B** HepG2 cell line treated with 0.22 mg/mL root extract. **C** HepG2 cell line treated with 0.30 stem extract. **D** HT-29 cell line treated with 0.1% DMSO. **E** HT-29 cell line treated with 1.13 mg/mL root extract. **F** HT-29 cell line treated with 1.50 mg/mL stem extract

**Fig. 3:**
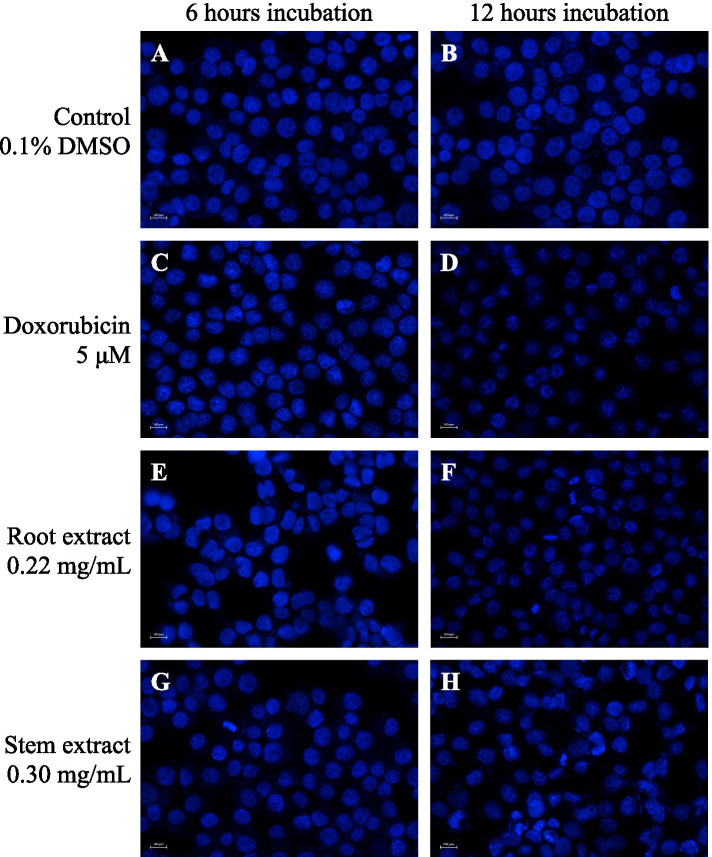
Fluorescent microscopic analysis of extracts treated HepG2 cell line stained with Hoescst 33,342. **A** and **B** are 0.1% DMSO treated HepG2 cell line at 6 and 12 h, respectively. **C** and **D** are 5 μM doxorubicin treated HepG2 cell line at 6 and 12 h, respectively. **E** and **F** are 0.22 mg/mL root extract treated HepG2 cell line at 6 and 12 h, respectively. **G** and **H** are 0.30 mg/mL stem extract treated HepG2 cell line at 6 and 12 h, respectively

We used inverted microscope to observe the morphology of the extract treated HepG2 and HT-29 cell lines comparing with their negative control (0.1% DMSO). We found that the root and stem aqueous extracts treated HepG2 and HT-29 cell lines changed their morphology when comparing with untreated control cell lines. The morphologies including cell blebbing, shrinkage, and formation of late apoptosis liked cells were clearly observed in all 24 h extracts treated cell lines at their IC_50_ concentrations (Fig. 2). To prove our hypothesis on apoptosis, we then cultured HepG2 cell line, treated the cell line with extracts, and stained the cell line by Hoechst 33342 stain in cell culture chamber slide. The fluorescent microscopic analysis showed the nuclear condensation behavior of the extract treated HepG2 cell line at IC_50_ concentrations (0.22 mg/mL of root extract and 0.30 mg/mL of stem extract), but not in negative control untreated HepG2 cell line (0.1% DMSO) (Fig. 3).

### Antibacterial activity of the *Bauhinia strychnifolia* Craib aqueous extracts

We determined antibacterial activity of root and stem extracts against 4 important human gut ecosystem pathogens. Among these pathogens, *Bacillus cereus* and *Staphylococcus aureus* represented gram-positive bacteria while *Escherichia coli* and *Pseudomonas aeruginosa* represent gram-negative bacteria. The disc diffusion assay results obviously showed that the *Bauhinia strychnifolia* Craib root and stem extracts inhibited the growth of *Bacillus cereus* and *Staphylococcus aureus* but cannot inhibited the growth of *Escherichia coli* and *Pseudomonas aeruginosa* (see Additional file 1). Consequently, we determined the MIC values of root and stem aqueous extracts against *Bacillus cereus* and *Staphylococcus aureus*. The broth microdilution assay results showed the similar MIC values of all analyses at 128 μg/mL (Table 2).

**Table 2:** IZD values from the disc diffusion assay and MIC values from the broth microdilution assay

Bacteria	IZD (mm) (mean ± SEM)					MIC (μg/mL)		
	Root extract		Stem extract		Positive control^a^	Root extract	Stem extract	Positive control^b^
	2 mg	5 mg	2 mg	5 mg				
*Bacillus cereus*	11.6 ± 0.7	14.4 ± 1.2	10.4 ± 0.7	13.4 ± 1.2	19.2 ± 2.0	128	128	8
*Escherichia coli*	N/A	N/A	N/A	N/A	17.0 ± 1.6	ND	ND	ND
*Pseudomonas aeruginosa*	N/A	N/A	N/A	N/A	20.4 ± 1.9	ND	ND	ND
*Staphylococcus aureus*	11.2 ± 0.7	12.4 ± 0.7	9.2 ± 0.7	11.2 ± 0.7	31.0 ± 1.6	128	128	8

^a^ Positive control impregnated discs are ampicillin (10 μg/disc), ceftriaxone (30 μg/disc), chloramphenicol (30 μg/disc), and rifampicin (5 μg/disc) for *Escherichia coli*, *Pseudomonas aeruginosa*, *Bacillus cereus*, and *Staphylococcus aureus*, respectively. ^b^ Positive control are chloramphenicol and rifampicin for *Bacillus cereus*, and *Staphylococcus aureus*, respectively. N/A value is defined as zero. ND means not determined

### Antioxidant activity of *Bauhinia strychnifolia* Craib aqueous extracts

**Fig. 4:**
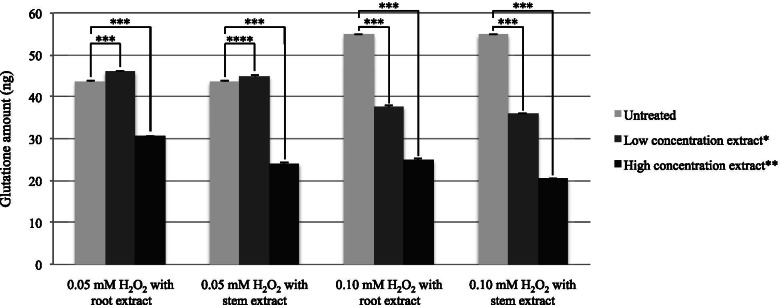
Cellular GSH amount of oxidative stressed HepG2 cells treated with different concentration of root and stem extracts. Untreated is 0 mg/mL for both root and stem extracts. *Low concentration extract is 0.30 mg/mL and 0.15 mg/mL for root and stem extracts, respectively. **High concentration extract is 0.60 mg/mL and 0.3 mg/mL for root and stem extracts, respectively. ***The pair is statistically significant with *p* < 0.001. ****The pair is statistically significant with *p* < 0.01

We first determined total antioxidant capacity of the *Bauhinia strychnifolia* Craib root and stem extracts using Cayman’s ABTS kit. We found that the root and stem extracts possessed total antioxidant capacity (Trolox equivalent) at 1.66 ± 0.99 and 2.51 ± 0.10 μmol/g extract (*p*-value < 0.001), respectively. Furthermore, we determined the cellular response of the root and stem extracts treated oxidative stressed HepG2 cells comparing with the untreated oxidative stressed cells using Abcam’s GSH + GSSG/GSH assay kit. We hypothesized that the extract treated oxidative stressed cells produced more GSH than the control oxidative stressed cells for scavenging of free radicals. The result confirmed the increasing of GSH production from 43.58 ± 0.17 ng in control 0.05 mM H_2_O_2_ oxidative stressed HepG2 cells to 46.00 ± 0.07 (*p*-value < 0.001) and 44.87 ± 0.20 (*p*-value < 0.001) ng in 0.3 mg root and 0.15 mg stem extract treated 0.05 mM H_2_O_2_ oxidative stressed HepG2 cells, respectively. However, at high concentration of H_2_O_2_ (0.1 mM) or high concentration of extracts (0.6 mg root extract or 0.3 mg stem extract), the extract treated H_2_O_2_ oxidative stressed HepG2 cells entered apoptosis phase and produced less GSH than the control H_2_O_2_ oxidative stressed HepG2 cells (*p*-value < 0.05) (Fig. 4).

### Major chemical constituents in the *Bauhinia strychnifolia* Craib root and stem aqueous extracts

**Fig. 5:**
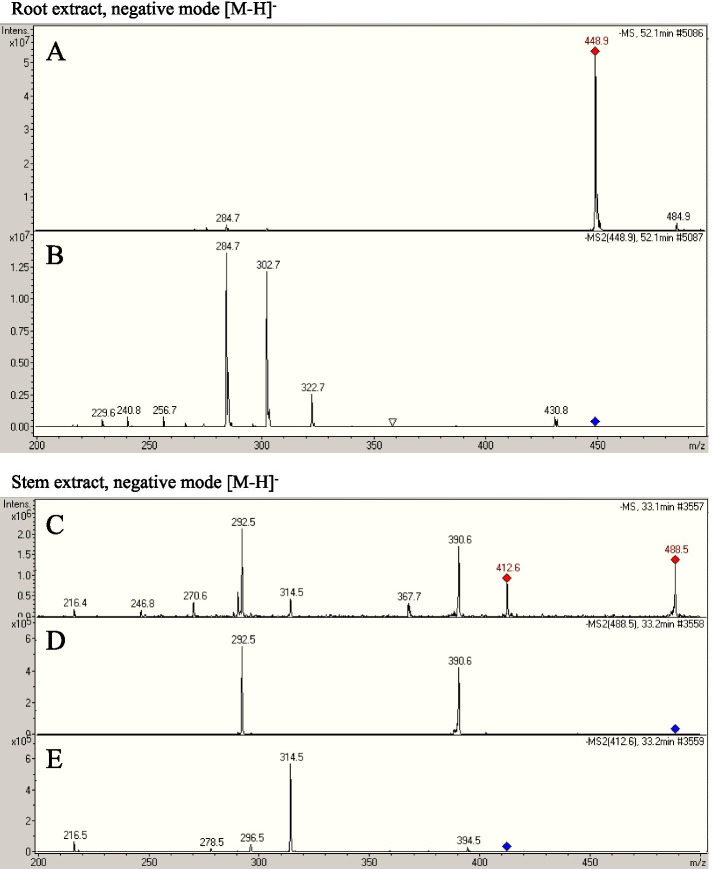
LC-MS analysis of root and stem extracts for elucidation of their major compositions. For root extract, (**A**) a major compound found at [M-H]^−^ 448.9 m/z and (**B**) its matched fragmented masses of 3,5,7,3′,5′-pentahydroxyflavanonol-3-O-α-L-rhamnopyranoside. For stem extract, (**C**) a major compound found at [M-H]^−^ 412.6 m/z and (**E**) its matched fragmented masses of β-sitosterol together with (D) unmatched fragmented masses of a unknown compound found at [M-H]^−^ 488.5 m/z

We would like to elucidate the chemical composition inside the root and stem aqueous extracts whether they have similar major compositions as the alcohol extracts or not. Using LC-MS analysis, we found that the *Bauhinia strychnifolia* Craib root and stem aqueous extracts had the similar major masses of compounds to the alcohol extracts in previous study [3]. We found the [M-H]^−^ mass of 3,5,7,3′,5′-pentahydroxyflavanonol-3-O-α-L-rhamnopyranoside at calculated 449 m/z (found 448.9 m/z) in root and β-sitosterol at calculated 413 m/z (found 412.6 m/z) in stem extracts. Moreover, there was another major [M-H]^−^ mass shown at 488.5 m/z representing an unknown compound found in *Bauhinia strychnifolia* Craib stem extract (Fig. 5).

## Discussion

Our results confirmed that both *Bauhinia strychnifolia* Craib root and stem aqueous extracts could inhibit the growth of HepG2 and HT-29 cell lines. In addition, we found that the root aqueous extract better inhibited the growth of the cell lines than stem aqueous extract. The results reflected the different compositions and/or ratio of chemicals, especially for antiproliferative compounds, between the parts of the plant. The results were similar to the analysis of anticancer activity of *Bauhinia strychnifolia* Craib dichloromethane, ethanol, hexane, and aqueous extracts in 2012. Furthermore, 5 pure compounds were isolated from *Bauhinia strychnifolia* Craib ethanol fraction and identified for their anticancer activity in 2013. The studies confirmed that the extracts and isolated pure compounds inhibited the growth of A549, HT-29, HeLa, KB, KB3–1, MCF-7, MDA-MB-231, and SW480 cell lines; however, they did yet describe the mechanism of inhibition [3, 4]. In addition to MTT assay, our phase contrast microscopic analysis together with Hoechst 33342 staining suggested that *Bauhinia strychnifolia* Craib root and stem aqueous extracts would likely inhibit the growth of HepG2 and HT-29 cell lines through apoptosis pathway. Accordingly, the administration of *Bauhinia strychnifolia* Craib root and stem decoction could really aid cancer patients with the hepatocellular carcinoma and colon adenocarcinoma as claimed in Thai traditional medicine.

In some cancer patients, especially with the colon cancer wound infected by opportunistic pathogens, they could suffer more than the others [11]. There were some evidences showing that the administration of *Bauhinia strychnifolia* Craib decoction could reduce diarrhea causing by gut ecosystem pathogens [2]. Accordingly, we determined the antibacterial activity of the extracts against 4 important gut pathogens including 2 g-positive bacteria *Bacillus cereus* and *Staphylococcus aureus* and 2 g-negative bacteria *Escherichia coli* and *Pseudomonas aeruginosa*. We found that the *Bauhinia strychnifolia* Craib root and stem aqueous extracts could kill the gram-positive bacteria but not killed gram-negative bacteria. There was no previous study on the antibacterial activity analysis of *Bauhinia strychnifolia* Craib; our study was the first report for the selective inhibition of root and stem aqueous extracts against gram-positive bacteria *Bacillus cereus* and *Staphylococcus aureus*. Considering MIC values, the inhibition activity of root and stem aqueous extracts (128 μg/mL) against *Staphylococcus aureus* is comparable with known diarrheal treating plants such as polyphenol extracts of wild blackberry (*Rubus fruticosus*, 125 μg/mL), blackthorn (*Prunus spinosa L.*, 125 μg/mL) and European cornel (*Cornus mas*, 125 μg/mL) [12] but better than aqueous extracts of lime (*Citrus aurantifolia*, 256 μg/mL) [13] and green tea (*Camellia sinensis*, 400 μg/mL) [14]. Our results supported the hypothesis that the administration of *Bauhinia strychnifolia* Craib decoction would help reducing diarrhea causing by the gram-positive pathogens.

We noticed that hangover remedy was one of the major uses of *Bauhinia strychnifolia* Craib decoction in Thai traditional medicine [3, 5–7]. The detoxification processes would be related with antioxidation activity of the antioxidant compounds or cellular free radical scavenging pathway stimulator existed in the decoction. We performed ABTS and GSH antioxidant assays to confirm the presence of the antioxidants in *Bauhinia strychnifolia* Craib root and stem aqueous extracts. The results from our ABTS assay suggested that the root and stem aqueous extracts had comparable total antioxidant capacity with several vegetables and fruits analysed in 2007. *Bauhinia strychnifolia* Craib root and stem aqueous extracts had Trolox equivalent values (1.66 ± 0.99 and 2.51 ± 0.10 μmol/g extract for root and stem extracts, respectively) similar to red onion (1.76 μmol/g) and tomato (1.17 μmol/g), 3 times higher than spinach (0.28 μmol/g), but 3 times less than orange (5.06 μmol/g) [15]. After treating oxidative stressed HepG2 cell line with the root and stem aqueous extracts and determining GSH production inside the cell, we found that the low concentration extracts treated HepG2 cell line produce higher GSH than the untreated oxidative stressed HepG2 cell line. However, the oxidative stressed HepG2 cell line entered apoptosis after we treated them with high concentration extracts. Consequently, the high concentration extracts treated oxidative stressed cells produced lower amount of GSH than the untreated cells. At this point, we could assume that treating HepG2 cell line with *Bauhinia strychnifolia* Craib root and stem aqueous extracts at low concentration stimulating the cellular free radical scavenging pathway via production of GSH similar to several previous studies [16–20]. Together, it might be the reason why we would be able to use *Bauhinia strychnifolia* Craib decoction as hangover remedy.

According to the study in 2013, 5 major metabolites from ethanol fraction of *Bauhinia srtychnifolia* Criab were isolated, purified, and determined for their anticancer activity against several cell lines. We suspected that some of these metabolites might be presented in the *Bauhinia strychnifolia* Craib root and stem aqueous extracts. We did LC-MS analysis to confirm the presence of the 5 compounds. For root extract, we found 3,5,7,3′,5′-pentahydroxyflavanonol-3-O-α-L-rhamnopyranoside, one among the 5 isolated compounds, as a major metabolite. The metabolite had very high potent cytotoxicity against HeLa (IC_50_ 0.0692 μg/mL), HT-29 (IC_50_ 0.00217 μg/mL), KB (IC_50_ 0.00054 μg/mL), and MCF-7 (IC_50_ 0.0585 μg/mL) cell lines [3]. A study in 2019 suggested that the compound could inhibit the activity of α-glucosidase with IC_50_ of 0.98 mg/mL. According to the bioactivity, the compound was proposed as a potent antidiabetic drug [7]. Not only that, a molecular docking study in 2020 revealed that the compound could bind and inhibit cyclin-dependent protein kinase 2/CDK-2 inducing the cell lines to enter apoptosis phase [21]. Obviously, *Bauhinia strychnifolia* Craib root aqueous extract, which contain 3,5,7,3′,5′-pentahydroxyflavanonol-3-O-α-L-rhamnopyranoside as a major metabolite in our study, should be able to exhibit the antiproliferative activity against both HepG2 and HT-29 cell lines.

For stem extract, we found β-sitosterol as a major compound together with an unknown metabolite [3]. For β-sitosterol, it is a regarded as a safe nutritional supplement that can be sold commercially in the market [22]. It was recognized to possess several bioactivities including anti-nociceptive [23], anxiolytic & sedative effects [24], analgesic [25], immune modulatory [26], antimicrobial [27], anti-inflammatory [28], protective effect against non-alcoholic fatty liver disease [29], lipid lowering [30], hepatoprotective [31], protective effect on respiratory diseases [32], wound healing [33], antidiabetic [34], antioxidant [35], and anticancer [36]. As an anticancer, it could inhibit the growth of HeLa, HT-29, KB, and MCF-7 cell lines with moderate IC_50_ values ranging from 0.099 to 1.49 μg/mL [3]. We found that *Bauhinia strychnifolia* Craib stem aqueous extract exhibited moderate free radicals scavenging activity similar to several other plants that have β-sitosterol as major component including fir, purple yam, water pepper, nutmeg, white mustard, anise, coriander, and caraway [37]. Theoretically, β-sitosterol should reverts the GSH/oxidized GSH ratio value by GSH turnover (reduction of oxidized GSH back to GSH). However, a study in 2005 revealed that β-sitosterol is a key stimulator involving in a protective intracellular antioxidant defense mechanism. It modulates antioxidant enzymes in estrogen receptor/PI3-kinase-dependent pathway, thus increasing GSH production in the β-sitosterol treated macrophage cells [38]. In 2011, streptozotocin-induced diabetic rats were treated with β-sitosterol. The increasing of GSH level in pancreatic tissue was detected similar to the result from our study [39]. Moreover, a recent study in 2017 also suggested that β-sitosterol could increase production of GSH and inhibited cholinesterase in sub-strain of transgenic Alzheimer’s disease mice [40]. The production of GSH detected in our study could be a result from free radical scavenging activity and/or stimulation of cellular defence mechanism by β-sitosterol similar to the previous studies.

## Conclusion

The decoction prepared from root and stem of *Bauhinia strychnifolia* Craib could inhibit the growth of hepatocellular carcinoma and colon adenocarcinoma cell lines with impressed IC_50_ values. In addition, they could also inhibit the growth of gram-positive bacterial pathogens such as *Staphylococcus aureus* and *Bacillus cereus*. Moreover, the ABTS and GSH assays confirmed the existence of antioxidant and/or cellular free radical protective stimulant inside the root and stem aqueous extracts. Our LC-MS analyses suggested that the root and stem decoctions contained 3,5,7,3′,5′-pentahydroxyflavanonol-3-O-α-L-rhamnopyranoside and β-sitosterol, which exhibit potent anticancer and antioxidant bioactivities. Altogether, the results of our analyses supported that administrating *Bauhinia strychnifolia* Craib root and stem decoctions would give beneficial effects on both colon and liver ecosystem as the claimed in Thai traditional medicine.

## Supplementary Information

**Additional file 1.**

**Additional file 2.**

## Data Availability

The datasets used and/or analysed during the current study are available from the corresponding author on reasonable request.

## Abbreviations

ABTS: 2,2′-azino-di 3-ethylbenthiazolinesulfonate
ANOVA: Analysis of variance
ATCC: American Type Culture Collection
DMEM: Dulbecco’s modified eagle medium
DMSO: Dimethyl sulfoxide
FBS: Fetal bovine serum
GSH: Glutathione
GSSG: Glutathione disulfide
IC_50_: The half maximal inhibitory concentration
IZD: Inhibition zone diameter
LC-MS: Liquid chromatography–mass spectrometry
MHB: Mueller-Hinton broth
MIC: Minimal inhibitory concentration
MTT: 3-(4,5-dimethylthiazol-2-yl)-2,5-diphenyl tetrazolium bromide
N/A: Not available or zero
NA: Nutrient agar
NB: Nutrient broth
ND: Not determined
NSS: Normal saline solution
SEM: Standard error of mean
SRB: Sulforhodamine B
SSA: Sulfosalicylic acid solution
