# Supplementary material for: Antiproliferative, antibacterial, and antioxidant activities of Bauhinia strychnifolia Craib aqueous extracts in gut and liver perspective
Source: BMC Complement Med Ther. 2021 Nov 4;21:276. doi: 10.1186/s12906-021-03448-2 (PMC8567622; doi:10.1186/s12906-021-03448-2)
Supplement: Supplementary file 1 — Additional file 1. [file 12906_2021_3448_MOESM1_ESM.pdf]

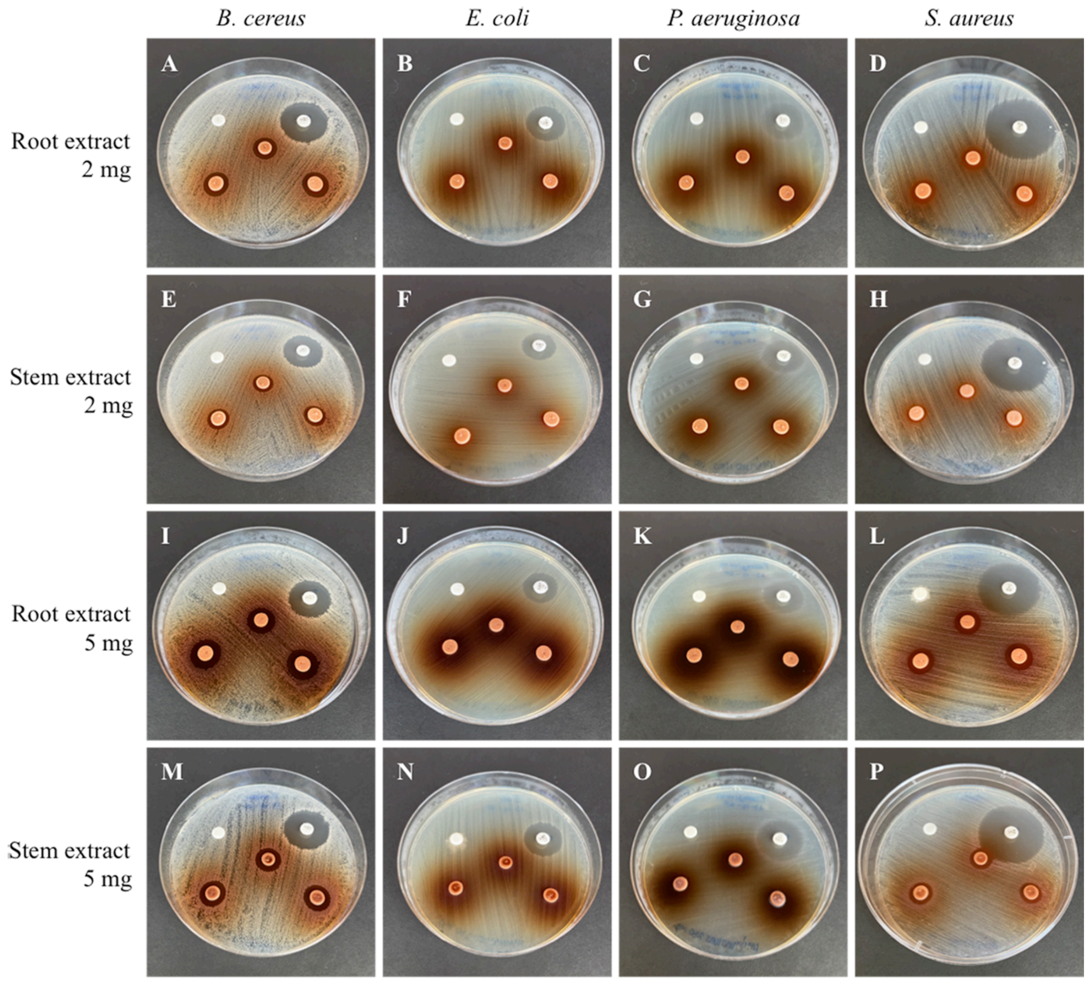

Additional file 1. Disc diffusion assay of the root and stem extract against gut pathogenic bacteria strains. (A, B, C, and D) are 2 mg impregnated root extract against 4 bacteria. (E, F, G, and H) are 2 mg impregnated stem extract against 4 bacteria. (I, J, K, and L) are 5 mg impregnated root extract against 4 bacteria. (M, N, O, and P) are 5 mg impregnated stem extract against 4 bacteria. DMSO impregnated discs were used as negative control while ampicillin (10 µg/disc), ceftriaxone (30 µg/disc), chloramphenicol (30 µg/disc), and rifampicin (5 µg/disc) were as positive control for *Escherichia coli*, *Pseudomonas aeruginosa*, *Bacillus cereus*, and *Staphylococcus aureus*, respectively
