# Supplementary material for: Antiproliferative, antibacterial, and antioxidant activities of Bauhinia strychnifolia Craib aqueous extracts in gut and liver perspective
Source: BMC Complement Med Ther. 2021 Nov 4;21:276. doi: 10.1186/s12906-021-03448-2 (PMC8567622; doi:10.1186/s12906-021-03448-2)
Supplement: Supplementary file 2 — Additional file 2. [file 12906_2021_3448_MOESM2_ESM.pdf]

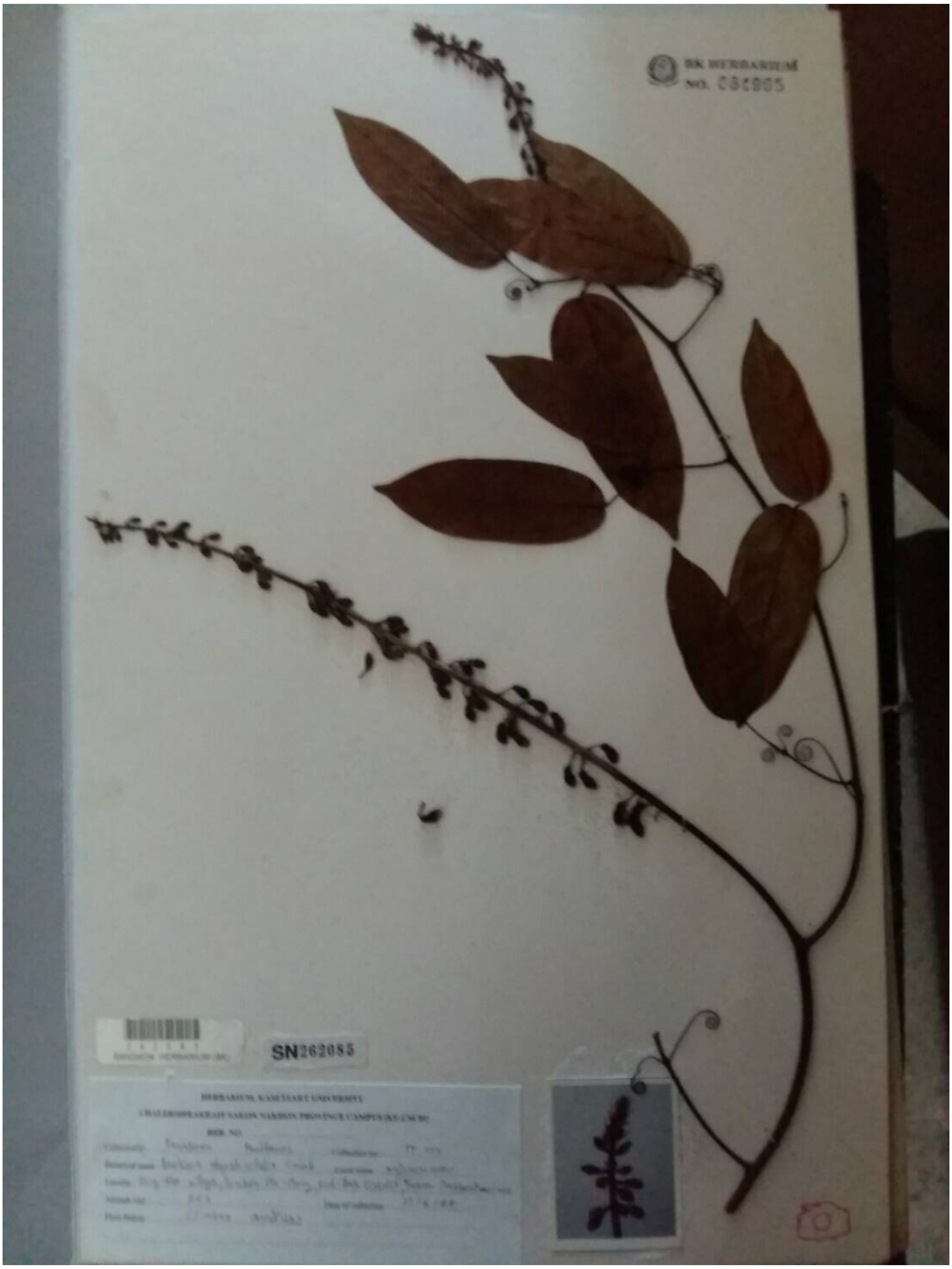

Additional file 2. Voucher specimen number BK No. 084965 identified as *Bauhinia strychnifolia* Craib and kept at BK Herbarium (Botanical Section, National Department of Agriculture, Thailand). The harvester, Nirun Vipunngeun (Department of Pharmacognosy, Faculty of Pharmacy, Rangsit University), compared and harvested the cultivated *Bauhinia strychnifolia* Craib plant materials under the

national guideline of the BK Herbarium from our own Rangsit University’s cultivation field.
